# *ROS-1* Fusions in Non-Small-Cell Lung Cancer: Evidence to Date

**DOI:** 10.3390/curroncol29020057

**Published:** 2022-01-28

**Authors:** Sébastien Gendarme, Olivier Bylicki, Christos Chouaid, Florian Guisier

**Affiliations:** 1 INSERM, IMRB (Clinical Epidemiology and Ageing Unit), University Paris Est Créteil, F-94010 Créteil, France; christos.chouaid@chicreteil.fr; 2 Pneumology Department, Centre Hospitalier Intercommunal de Créteil, 40, Avenue de Verdun, F-94010 Créteil, France; 3 Respiratory Disease Unit, HIA Sainte-Anne, 2, Boulevard Saint-Anne, F-83000 Toulon, France; bylicki.olivier@yahoo.fr; 4 Department of Pneumology, Rouen University Hospital, 1 Rue de Germont, F-76000 Rouen, France; florian.guisier@chu-rouen.fr; 5 Clinical Investigation Center, Rouen University Hospital, CIC INSERM 1404, 1 Rue de Germont, F-76000 Rouen, France

**Keywords:** ROS-1 protein, protein tyrosine-kinase receptors, lung cancers, non-small-cell lung cancer

## Abstract

The *ROS-1* gene plays a major role in the oncogenesis of numerous tumors. *ROS-1* rearrangement is found in 0.9–2.6% of non-small-cell lung cancers (NSCLCs), mostly lung adenocarcinomas, with a significantly higher rate of women, non-smokers, and a tendency to a younger age. It has been demonstrated that *ROS-1* is a true oncogenic driver, and tyrosine kinase inhibitors (TKIs) targeting ROS-1 can block tumor growth and provide clinical benefit for the patient. Since 2016, crizotinib has been the first-line reference therapy, with two-thirds of the patients’ tumors responding and progression-free survival lasting ~20 months. More recently developed are ROS-1-targeting TKIs that are active against resistance mechanisms appearing under crizotinib and have better brain penetration. This review summarizes current knowledge on *ROS-1* rearrangement in NSCLCs, including the mechanisms responsible for *ROS-1* oncogenicity, epidemiology of *ROS-1*-positive tumors, methods for detecting rearrangement, phenotypic, histological, and molecular characteristics, and their therapeutic management. Much of this work is devoted to resistance mechanisms and the development of promising new molecules.

## 1. Introduction

Lung cancer represents the leading cause of cancer deaths worldwide, with more than 1.8 million deaths in 2020 [1]; 85% are non-small-cell lung cancers (NSCLCs) and 25% of them harbor oncogenic alterations that can be targeted by therapy. That is the case for patients whose tumors are positive for proto-oncogene tyrosine-protein kinase-1 (*ROS-1*; *c-Ros* oncogene-1)-gene fusion. Identification of that translocation makes patients eligible for targeted therapy. Prospective phase I/II trial results have shown the efficacy of crizotinib, that is a tyrosine-kinase inhibitor (TKI) targeting ROS, anaplastic lymphoma kinase protein (ALK) or mesenchymal-to-epithelial transition (MET) protein, and is now recommended as first-line therapy [2,3,4]. Unfortunately, despite initial responses, *ROS-1*-positive NSCLCs develop resistances to crizotinib, allowing tumor progression, notably brain metastases. More recently devised new molecules are active against crizotinib resistances and have good brain penetration.

This present review is an update of those previous reviews [5,6] and describes our current knowledge of *ROS-1* rearrangement in NSCLCs, including their diagnostic modalities, epidemiology, and characteristics, and the development of diverse molecules targeting ROS-1 and the identified resistance mechanisms.

## 2. *ROS-1* Gene

The *ROS-1* gene was discovered in the 1980s as the product of the avian sarcoma virus RNA UR2 (University of Rochester) [7]. This gene codes for 2347 amino acids that form a transmembrane protein sharing structural characteristics with the family of insulin receptors and ALK. The ROS-1 protein is composed of an extracellular domain containing a hydrophobic segment allowing transmembrane passage and an intracellular component containing a tyrosine-kinase domain with a terminal carboxyl [8]. Its physiological role is poorly understood but study results suggest that wild-type ROS is involved during embryonic development as an initiator of signaling events for the differentiation of epithelial tissues [9]. Early during the 2000s, the proto-oncogene role of ROS was first identified in brain tumors. A microdeletion in chromosome 6q21 is responsible for *ROS-1* fusion with a new fused-in-glioblastoma (*FIG*) gene that is responsible for *ROS-1* overexpression and production of signals abnormally activating the tyrosine-kinase pathway, conferring its proto-oncogene role [10]. In mouse models, FIG–ROS-1 transcript expression induces tumorigenesis and treatment with a small molecule, TAE684, inhibits growth of Baf3 cells overexpressing short and long isoform of *FIG-ROS-1*, thereby defining *ROS-1* as an oncogenic “driver”.

The *ROS-1* proto-oncogene role in lung cancer was first reported in 2007 by Rikova et al., who identified two other protein fusion transcripts: a transmembrane solute transporter (SLC34A2) and a type-2 transmembrane protein (CD74) [11]. Since then, improved sequencing techniques have enabled the discovery of increasing numbers of fusion partners [12,13], whose proto-oncogene roles in numerous cancers is now clearly established [14].

*ROS-1* plays a major role in the activation of several signaling pathways associated with differentiation, proliferation, cell growth, and survival. *ROS-1* rearrangement, by forming phosphotyrosine-recruitment sites in the terminal tail of ROS, causes kinase-activity deregulation of the protein and abnormal activation of signaling pathways, mediated by tyrosine-phosphatase tumor-suppressor SHP1/SHP2, pro-mitotic protein extracellular signal-regulated kinase (ERK1/2), insulin-receptor substrate (IRS-1), phosphatidylinositol 3-kinase (PI3K) pathway, protein kinase B (AKT), mitogen-activated protein kinases (MAPKs), signal transducer and activator of transcription (STAT3), and VAV3 [15]. However, the ligand binding to its recruitment site initiating ROS-protein activation remains unknown. Moreover, study results suggest that activation of the pathways could depend on the fusion transcript. Thus, in the case of *CD74–ROS-1* fusion, the role played by phosphorylation of the E-Syt-1 (extended synaptotagmin-like) protein could confer the tumor cell with greater metastatic and invasive potential [16].

## 3. Epidemiology, Clinical, and Histological Characteristics

ROS-1 rearrangement is present in approximately 0.9–2.6% of NSCLCs [13,17,18,19,20]. Like ALK rearrangements, it is more frequent in young subjects, women and never-smokers [13,17,18,19,20]. ROS-1-positive NSCLCs are predominantly lepidic, acinar, or solid adenocarcinomas, with more than 90% expressing thyroid transcription factor-1 (TTF1), diagnosed at an advanced stage (stage III–IV), with a higher frequency of brain metastases [20,21,22,23,24]. More rarely, *ROS-1* rearrangement is found in other histological subtypes, e.g., squamous-cell carcinomas, pleomorphic carcinomas or large-cell carcinomas [13,23,25,26,27]. Histological examination of these tumors mainly finds a solid architecture with round nuclei containing macronucleolus, “signet-ring cell”, and a close relationship with adjacent bronchioles [25]. Imaging shows metastatic lymph-node tropism, often reported with less frequent extrathoracic metastatic sites than for ALK rearranged or epidermal growth factor receptor (EGFR)-mutated NSCLCs [28]. Miliary forms of lung metastases have also been described [29]. Several authors reported a heightened thromboembolic risk of ROS-1-rearranged tumors compared with NSCLCs harboring non-rearranged ROS-1 [30], and even rarer cases of thrombotic microangiopathies [31] and disseminated intravascular coagulation [32]. Although the underlying mechanisms remain to be elucidated, interaction between extracellular carcinoid mucins secreted into the bloodstream and platelet (P-) and leukocyte (L-) selectins could trigger platelet activation and the formation of microthrombi responsible for thromboembolic events [33].

## 4. Molecular Characteristics

### 4.1. Fusion Partners

ROS-1 rearrangement occurs at a breakpoint in the *ROS* gene at the 5′ end of exons 32, 34, 35, or 36, or introns 31 or 33 [14,34]. The most frequently seen fusion partners (Table 1) are *CD74* (38–54%), *EZR* (13%–24%), *SDC4* (9–13%), and *SLC34A2* (5–10%) [34,35]. However, improved DNA- and RNA-sequencing techniques have enabled identification of numerous fusion partners, including *CCCKC6, TFG, SLMAP, MYO5C, FIG, LIMA1, CLTC, GOPC, ZZCCHC8, CEP72, MLL3, KDELR2, LRIG3, MSN, MPRIP, WNK1, SLC6A17, TMEM106B, FAM135B, TPM3,* and *TDP52L1* [36]. The prognostic role of fusion partners is still being debated. The results of several studies showed that the presence of CD74–ROS-1 rearrangement was associated with longer progression-free survival (PFS) and overall survival (OS) than non-*CD74–ROS-1* rearrangement [36]. However, that association with survival was not found in other studies [34].

### 4.2. Oncogenic Co-Mutations

About 36% of *ROS-1*-positive NSCLCs have oncogenic co-mutations [48]. Their frequencies are lower compared to cancers with non-rearranged ROS-1 [35] and are usually mutually exclusive with Kirsten rat-sarcoma viral oncogene (KRAS), EGFR, and ALK mutations [49]. However, rare co-mutations with other so-called driver oncogenes have been reported [50,51]. Lambros et al., reported 15 cases of co-mutations between ROS-1 and EGFR with nine exon-19 deletions, one exon-20 insertion, and five L858R mutations. A first-line EGFR TKI obtained a tumor response or stability for 80% of the patients. ROS-1 rearrangement appeared in one tumor as a resistance mechanism under EGFR-TKI and crizotinib was able to obtain a partial response [52]. A low percentage of co-mutations involving EGFR was reported in several papers [48]. Extremely rare ALK and ROS-1 co-mutations have also been described [50,53]. Given that both of these mutations are sensitive to crizotinib, the treatment choice is less problematic than for other co-mutations. The NSCLCs of all reported patients who had benefited from systemic treatment responded to crizotinib.

KRAS co-mutations can be present at diagnosis or progression. The scarce data available in this setting render evaluation of crizotinib sensitivity difficult. Based on six patients with KRAS–ROS co-mutation at diagnosis, a tumor response was obtained for 1; but not in KRASG13D- or G12V-mutation carriers [48,49].

ROS-1-positive NSCLCs with MET amplification are extremely rare. Tang et al., explored the genetic co-alterations involving ALK, ROS-1, RET, and MET in a series of 15 patients and found one ROS-1–MET co-mutation but they provided no treatment information [54]. One reported case of ROS-1–c-MET co-mutations experienced capmatinib (TKI anti-MET) failure followed by a 11 months crizotinib benefit [55]. In contrast, Zeng et al., described a patient with ROS-1-positive NSCLC and MET amplification whose tumor progressed 1.5 months after starting crizotinib [56].

Co-mutations between ROS-1–BRAF (v-RAF murine sarcoma viral oncogene homolog B) have been published, but without any information on anti-ROS-1 or anti-BRAF treatments in this context [24,48]. To the best of our knowledge, *ROS-1* co-mutation with rearranged-during-transfection translocation (*RET*) or human epidermal growth factor receptor 2 (*HER2*) have not been described to date. Other non-targetable co-mutations also have a prognostic role, for example, tumor protein 53 (*TP53*), which was associated with shorter survival in one study [57]. In another study, Zeng et al. [56] found that patients whose NSCLCs had an exclusive *ROS-1* fusion lived longer without progression than those whose tumors carried a *ROS-1* fusion with co-mutations (15.5 vs. 8.5 months, respectively; *p* = 0.0213).

## 5. Techniques of ROS-1 Detection

It is now recommended that *ROS-1* rearrangement be sought in all metastatic lung carcinomas, regardless of clinical characteristics. This search should also be considered in tumors with mixed histologies, when an adenocarcinoma component cannot be excluded.

For metastatic squamous-cell tumors, ROS-1 status can be assessed for never-smokers [57]. Immunohistochemistry (IHC) is used as a screening technique but positive or questionable results require confirmation by fluorescence in situ hybridization (FISH) or next-generation sequencing (NGS) [57]. Running NGS at diagnosis is also an alternative [58].

IHC ROS-1-labeling is located in the cytoplasm but can vary depending on the fusion partner [59]. Three commercialized anti-ROS-1 antibodies are available: clone D4D6 (Cell Signaling Technology, Danvers, MA, USA), which is used in clinical studies; clone SP384 (Roche, Ventana, AZ, USA); and clone 1A1 (Origene, Rockville, MD, USA) [60]. All those clones have high sensitivity (90–100%), compared to FISH and NGS. However, unlike ALK IHC, ROS-1 specificity is variable, ranging from 70% to 90% [61], and depends on the clone used [62] and the positivity threshold applied [63,64]. Thus, almost a third of the patients with IHC-positive tumors do not have *ROS-1* rearrangement [48,65]. The IHC false-positives are more frequent for lepidic and/or acinar EGFR-mutated adenocarcinomas [66]. Hence, it is now recommended that IHC-positivity and questionable ROS-1 results be confirmed with another technique: FISH, reverse transcription quantitative polymerase chain reaction (RT-qPCR) or NGS.

FISH is the gold standard to diagnose *ROS-1* rearrangements, using a dual probe (3′ and 5′) break-apart design. The sample is considered positive when more than 15% of the cells show separation of both 3′ and 5′ probes or a unique 3′ signal (centromeric) [65]. More than 50 tumor cells must be present to validate a positive finding. This technique has a relatively high price tag, technical difficulties (sufficient amount of tumor cells), and is operator-time consuming. At present, probes able to search simultaneously for *ALK* and *ROS-1* rearrangements, such as Vysis ALK/ROS-1 Dual Break Apart Probe Kit (RUO)) or FlexISH ALK/ROS-1 DistinguISH Probe Zytvision, require less tumor material and can be run on cytological samples [67,68]. IHC and FISH correspondence is high, but discordance between IHC and FISH is possible [69].

Notably, two situations can have major clinical consequences: (1) positive IHC-labeling of ROS-1 and negative FISH attributable to the presence of another oncogenic driver mutation, whose diagnosis is rectified by RT-PCR or NGS [70]; or (2) a false-negative FISH for certain fusion partners, primarily *GOPC–ROS-1* or *EZR–ROS-1* [71]. In the latter fusion, deletion of the 5′ end of *ROS-1* and unique 3′-probe-binding in FISH are often found [72]. Pertinently, authors of a recent study comparing IHC of ROS-1 clone SP384 to NGS also described IHC-positive expression in NSCLCs with rare *ROS-1* fusions (SQSTM1), *ROS-1* mutations and ROS-1 amplifications [38]. Thus, when a minor doubt persists after IHC and FISH, NGS should be run.

RT-qPCR uses primers specific to *ROS-1* fusion to confirm the presence of *ROS-1* rearrangement. However, the technique is not easily mastered, requiring different steps including RNA extraction, complementary DNA (cDNA) synthesis, quantitative PCR and analysis, which are all sources of variation [73]. This test’s performance is excellent, with 100% sensitivity and 85.1% specificity, according to one study [74].

NGS is able to identify all fusion partners, including new variants, and other oncogenic molecular alterations potentially targetable, by using tumor DNA or RNA [75]. Although DNA-based sequencing is able to detect rearrangements of fusion genes in intron regions, those genes often differ from messenger RNA (mRNA) fusions [76]. NGS can be run on tumor tissue or plasma [12]. However, its cost and time to results limit its use in routine clinical practice.

## 6. Treatment of ROS-1-Positive NSCLCs

Two TKIs have been validated as first-line therapy for ROS-1-positive NSCLCs: crizotinib, approved in 2016 by the Food Drug Administration (FDA) and the European Medications Agency (EMA), and entrectinib approved by the FDA in 2019 and EMA in 2020 [3,4].

### 6.1. Crizotinib

The results of the phase I PROFILE 1001 trial (NCT00585195), conducted on about 50 patients with metastatic ROS-1-positive NSCLCs, demonstrated crizotinib efficacy [2]. Given at a dose of 250 mg twice daily [2,77], the objective response rate (ORR) was 72% (95% CI 58–83%), the disease-control rate (DCR) was 90%, with median duration of response (DOR) at 24.7 (95% CI 15.2–45.3) months, and median PFS and OS, respectively, at 19.3 (95% CI 15.2–45.3) and 51.4 (95% CI 29.3–not reached) months. The most frequent adverse events were: vision disturbances (82%), diarrhea (44%), nausea (40%), peripheral edema (40%), constipation (34%), vomiting (34%), elevated transaminases (22%), fatigue (20%), and dysgeusia (18%), mainly grade 1 or 2. Toxicity never required crizotinib withdrawal.

Retrospective trials [78,79] and then prospective phase II trials [46,80,81,82] confirmed crizotinib efficacy in this population (Table 2). The phase III AcSé study, based on 37 patients, found an ORR of 47.2% [95% CI 30.4–64.5], with median PFS and OS, respectively, at 5.5 [95% CI 4.2–9.1], and 17.2 [95% CI 6.8–32.8] months. Those findings were less favorable than those of the PROFILE 1001 trial, probably because of a higher percentage of patients with Eastern Cooperative Oncology Group performance score (PS) = 2 (25% vs. 2%), who had been more heavily pre-treated [82]. The results of the European EUCROSS and METROS studies were closer to those obtained in the PROFILE 1001 trial, with respective ORRs of 70% [95% CI 51–85] and 65% [95% CI 44–82], with median PFS at 20 [95% CI 8.3–not reached], and 22.8 [95% CI 15.2–30.3] months. Factors associated with a poor prognosis were the presence of brain metastases, more than two metastatic sites and a TP53 mutation [41,46,81,82]. The presence of a CD74–ROS-1 fusion was associated with a more favorable prognosis in some studies [41] but had no impact according to others [36,83,84].

Despite crizotinib’s notable benefit in the management of ROS-1-positive NSCLCs, its poor brain penetration makes the central nervous system the primary site of progression under crizotinib (47% of patients). Moreover, given that 36% of patients with ROS-1-positive NSCLCs have brain metastases at diagnosis [22], the development of new ROS-1-targeting molecules with better cerebral penetration, such as entrectinib or lorlatinib, is justified.

### 6.2. Entrectinib

Entrectinib, a multikinase inhibitor targeting ROS-1, ALK or pan-tropomyosin-receptor kinase (TRK), penetrates the blood–brain barrier [85]. In vitro, its anti-ROS-1 activity is 40× more potent than that of crizotinib [86]. Two phase I/II trials showed entrectinib’s anti-tumoral activity and its good tolerance profile (Table 2) [87]. Its most frequent adverse events were: dysgeusia (41.4%), fatigue (27.9%), vertigo (25.4%), constipation (23.7%), diarrhea (22.8%), nausea (20.8%), and weight gain (19.4%). The results of the phase II basket study (STARTRK-2) confirmed entrectinib efficacy in 161 anti-ROS-1 treatment-naïve patients with ROS-1-positive NSCLCs, among whom 34.8% had brain metastases at diagnosis [88,89]. An updated analysis of the entire population found: ORR = 67.1% [95% CI 59.3–4.3], median PFS = 15.7 [95% CI 11.0–21.1] months, and median 1-year OS at 81% [95% CI 74–87]. For the 24 patients with measurable brain metastases at diagnosis, centralized assessment of the brain outcomes found ORR = 79.2% [95% CI 57.9–92.9], with median PFS at 12 months. The reported outcomes of 145 patients indicated non-clinically significant improvement of physical-function scores and quality of life (QOL; Health-Related Quality of Life scale) [90].

Based on those findings, entrectinib was approved in August 2019 by the FDA and the EMA accorded temporary authorization utilization in August 2020 to treat adults and children over 12 years old with ROS-1-rearranged NSCLCs at a dose of 600 mg/day [91].

### 6.3. Lorlatinib

Lorlatinib, a TKI active against ALK and ROS-1, was specifically developed to penetrate the blood–brain barrier by reducing the efflux mediated by P-glycoprotein-1. Lorlatinib (100 mg once daily) anti-tumor activity against ROS-1-positive NSCLCs was demonstrated in a phase I trial followed by a phase I/II trial on 61 TKI treatment-naïve (*n* = 21) or crizotinib-treated (*n* = 40) patients given another TKI (*n* = 8) (Table 2) [92,93,94]. TKI treatment-naïve patients obtained an ORR of 62% [95% CI 38–82], median PFS of 21 [95% CI 4.2–31.9] months and brain ORR of 64% [95% CI 31–89]. Median brain PFS was not reached, further supporting this molecule’s good efficacy against these metastases. For the crizotinib-pre-treated patients, ORR, median PFS and intracerebral objective response rate, respectively, were: 35% [95% CI 21–52], 8.5 [95% CI 4.7–15.2] months, and 50% [95% CI 29–71]. Grade-3 or -4 adverse events occurred, respectively, in 43% and 6% of the patients, with the most frequent being hypercholesterolemia (65%), hypertriglyceridemia (42%), peripheral edema (39%), peripheral neuropathies, (35%), altered cognitive functions (26%), weight gain (16%), and mood disorders (16%). Monitoring of plasma lorlatinib concentrations might achieve better control of adverse events without modifying anti-tumor efficacy [95]. Lorlatinib also achieved improved QLQ-C30 scale-evaluated QOL [96]. Lorlatinib efficacy seems to be even better when an ROS-1^K1991E^ or ROS-1^S1986F^ mutation is the mechanism of resistance against crizotinib; however, despite promising in vitro findings, activity appears to be more limited against the ROS-1^G2032R^ mutation, the principal resistance mechanism of ROS-1-positive NSCLC under crizotinib [97]. Notably, the NSCLCs of six patients harboring the ROS-1^G2032R^ mutation did not respond to lorlatinib in the phase I/II trial [94]. In addition, the tumor responses to lorlatinib after failure on entrectinib were also reported [98] but on too few patients to be meaningful. These exploratory observations must be confirmed to better specify the place of lorlatinib according to the tumor’s resistance profile to crizotinib.

### 6.4. Ceritinib

Ceritinib, a powerful ALK-specific inhibitor, exhibited antitumor efficacy and brain activity in patients with ALK-rearranged NSCLCs that progressed under crizotinib [99]. In vitro findings also suggested potent ceritinib inhibitory activity against ROS-1 [100]. A phase II study on 32 patients with ROS-1-positive NSCLCs, predominantly crizotinib-naïve (*n* = 30), treated with ceritinib (750 daily) achieved an ORR of 62% [95% CI 45–77], with median PFS = 9.3 [95% CI 0–22] months [100] (Table 2). In the subgroup of crizotinib-naïve patients, PFS lasted 19.3 [95% CI 1–37] months, comparable to those of other TKIs (entrectinib, lorlatinib). For the eight patients with brain metastases, the brain ORR was 63% [95% CI 31–86%]. Its tolerance profile was similar to those of other molecules, with 37% grade-3 adverse events. Those results do not make ceritinib a challenger for first-line crizotinib but it could have its place as a subsequent-line agent, according to the resistance profile and the progression sites.

### 6.5. Cabozantinib

Cabozantinib, a small molecule TKI, selectively targets MET, VEGFR-2, RET, ROS-1, and AXL, with good brain penetration. Pre-clinical and case-report findings suggest that cabozantinib can be effective in patients with NSCLCs resistant to other TKIs (crizotinib, entrectinib, ceritinib) by binding to ROS-1, despite the presence of resistance mutations, such as D2033N or G2032R [100,101]. Hence, cabozantinib could be a clinically pertinent agent to overcome crizotinib-resistance of ROS-1-positive NSCLCs [102]. An ongoing phase II trial (NCT01639508) is evaluating cabozantinib efficacy for this indication, with results expected in July 2022.

### 6.6. Brigatinib

Brigatinib, another ROS-1 inhibitor, has demonstrated anti-tumor activity against several crizotinib-resistance mechanisms [103]. One study assessed brigatinib efficacy and tolerance on eight patients ROS-1-positive NSCLCs, among whom 1 was TKI treatment-naïve and seven progressed post-crizotinib [104]. Their overall ORR to brigatinib was 37% and 29% for the seven post-crizotinib progressors. No grade-3 or -4 adverse event was reported. According to a case report, a tumor response was observed in a patient who had received several lines of anti-ROS-1 TKIs [105]. In vitro anti-tumor activity was suggested for NSCLC carrying the L2026M mutation [106] but no brigatinib efficacy against the G2032R mutation was found [103].

### 6.7. Other TKIs

In analogy to driver mutations, the current challenge of managing ROS-1-positive NSCLCs is the development of molecules with good brain penetration and activity against the main resistance mechanisms emerging under TKI(s). The ROS-1^G2032R^ resistance mutation is now the main target of new agents being developed.

Repotrectinib (TPX-0005) is a next-generation TKI that can target ROS-1, TRK, or ALK. Its properties enable its passage through the blood–brain barrier. This molecule was evaluated in pre-clinical models based on patients with ROS-1-positive NSCLCs [107]. The encouraging modeling results showed anti-tumor activity against brain ROS-1-positive metastases, treatment- ROS-1-positive, ROS-1-positive ceritinib-resistant, and ROS-1^G2032R^ NSCLCs. A clinical phase I/II trial (NCT03093116) is ongoing, with results expected in December 2022.

Taletrectinib (DS-6051B), a selective inhibitor of ROS-1 and neurotrophic TRAK (NTRAK), is capable of inhibiting crizotinib-resistant ROS-1-rearranged NSCLCs, including those harboring the G2032R mutation. Pre-clinical study results showed its efficacy in different mouse models derived from patients with ROS-1-positive NSCLCs [108]. Its in vitro activity against G2032R, L1951R, S1986F and L2026M mutations make it a particularly interesting molecule. In contrast, its activity is less certain against the D2033N mutation, for which it has higher minimal inhibitory concentrations (MICs). The clinical efficacy of this agent is being evaluated in two phase I trials, one American (U101, NCT02279433) and the other Japanese (Table 2) [109]. The former enrolled 46 patients and found 33% ORR for those with crizotinib-resistant tumors; the latter, conducted on 15 patients, reported 58.3% ORR for all patients and 66.7% for those crizotinib-naïve [109]. Brain activity was also observed but remains to be demonstrated. Thus, should efficacy and tolerance be confirmed, taletrectinib could play a promising role in the management of ROS-1-positive NSCLCs, notably those crizotinib-resistant.

Ensartinib (X-396) is a TKI with in vitro activity 10-fold higher than that of crizotinib against ALK [110]. Results of a recent phase II trial on ROS-1-positive NSCLCs (NCT03608007) showed modest efficacy of this molecule (ORR 27%, 95% CI 13.8–44.1) but interesting brain disease control in three out of four patients [111]. However, at present, use of this product in the management of NSCLCs harboring *ROS-1*-fusion genes has been retrograded.

### 6.8. What Is the Place of other Anti-Cancer Therapies?

Chemotherapy remains the reference second-line treatment after failure on crizotinib. Pemetrexed-based chemotherapy has potentially notable benefit against ROS-1-positive NSCLCs [112]. Anti-angiogenic agents could also play a role in combination with a TKI. Combining vascular endothelial growth factor (VEGF)-pathway blockade with a TKI targeting ROS-1 in vitro improved the anti-tumor effect compared to TKI alone [113]. According to a study based on 14 patients, the crizotinib–bevacizumab combination achieved efficacy against ALK-positive, ROS-1-positive or MET-amplified tumors with 58.3% ORR and an acceptable tolerance profile. Three patients developed hepatic toxicity or hemoptysis that required treatment discontinuation. Other TKI–anti-angiogenic agent combinations also yielded results of interest. Clinical trials on different combinations, such as BOOSTER (osimertinib–bevacizumab), could provide some answers about the potential benefits of this combined therapy [114].

The benefit of immunotherapy for ROS-1-positive NSCLCs, notably against crizotinib-resistant tumors, is unclear. In vitro and mouse models demonstrated that ROS-1 plays a role and modulates programmed death protein ligand-1 (PD-L1) expression via activation of MEK–ERK- and ROS-1–SHP2-signaling pathways [115]. In clinical practice, most ROS-1-positive tumors do not express PD-L1 and have a low mutation burden [115]. However, concerning other mutation drivers such as ALK or EGFR that apparently do not benefit from immunotherapy, the ORR for ROS-1-positive NSCLCs under the latter was 13–17% [116] and reached 83% in combination with chemotherapy but on small sample sizes [117]. Choudhury et al. [115] found no PD-L1–expression difference between responders and non-responders. Immunotherapy could also find a place in the therapeutic sequence for tumors resistant to several TKI lines combined with chemotherapy. Special attention should be paid to the toxicity risk of sequential immunotherapy–TKI treatments [115].

## 7. ROS-1-Positive NSCLC Resistance Mechanisms

### 7.1. Under Crizotinib

Crizotinib treatment of ROS-1-positive NSCLCs controls the disease for a median duration of 19 months before the tumor develops crizotinib-resistance mechanisms and tumor progression is observed. These resistance mechanisms are multiple: (1) appearance of a punctual mutation in the ROS-1–kinase domain and induces a modification of the binding site; (2) activation of other signaling pathways; and (3) phenotypic changes (mesenchymal-to-epithelial transition, transformation into small cells) [118,119].

#### 7.1.1. Punctual Mutation in the Kinase-Binding Domain (40–55%)

ROS-1–kinase-domain modification is the crizotinib-resistance mechanism (Table 3). First reported in 2013 [120], the G2032R mutation is the most frequent and represents 33%–41% of resistance mechanisms; it occurs in the solvent-front region of the ATP-binding site [120]. In in vitro studies, ROS^G2032R^ increased TWIST1 expression and led to mesenchymal-to-epithelial transition of cells and cell migration [121,122]. It induced resistance to several TKIs (crizotinib, ceritinib, entrectinib, and lorlatinib) via modification of the binding site and steric hindrance. At present, new-generation TKIs are being developed to target that mutation, with promising results obtained with repotrectinib and topotrectinib. Cabozantinib and foretinib have also shown anti-ROS^G2032R^ activity [102].

Other frequent mutations, in order of decreasing rates, are: D2033N (2.4–6%), S1986Y/F (2.4–6%), L2026M (1%), L2155S (1%), L1951R (1%), and S1886 (1%) [102]. ROS^D2033N^ induces a modification in the ATP-binding–site pocket and modified electrostatic interactions necessary for binding to anti-ROS-1 TKIs. In vitro, that mutation led to resistance to crizotinib, entrectinib, and ceritinib, while lorlatinib, repotrectinib, and cabozantinib remained sensitive. ROS-1^S1986F^ confers resistance to crizotinib, entrectinib and ceritinib by changing the position of the glycine-rich loop at the αC helix end [123]. The resistance profiles of other mutations are given in Table 3.

#### 7.1.2. Activation of Other Signaling Pathways

Crizotinib-resistance can also be linked to activation of other signaling pathways located after the ROS-1-protein kinase. Activation of these mechanisms can engender: (1) ROS-1 stimulation of signaling pathways resistant to inhibition by TKIs; (2) acquisition of new mutations or amplifications at the level of other proto-oncogene pathways. In the former case, SHP2 activation of the mitogen-activated protein kinase (MAPK) MEK/ERK pathway conferred TKI-resistance. In vitro, concomitant administration of SHP2 and TKI inhibitors better inhibited tumor growth and constitutes a promising avenue of research [125]. Activation of other signaling pathways, such as KIT, PI3K, and EGFR, has also been reported [34]. In the second case, mutations of ALK [125], BRAF [126], KRAS, neuroblastoma rat-sarcoma viral oncogene (NRAS) [34], and MET [127] genes and MET amplifications [128] have been described. A BRAFV600E mutation was found in two patients [78]; treatment with dabrafenib and trametinib was started, but both died shortly after starting therapy. For the MET mutation, tumor control was short-lived under cabozantinib [78]. For MET amplification, the patient died before having received an anti-MET-specific agent [127].

#### 7.1.3. Phenotype Change

Phenotype transformation into a small-cell carcinoma was described as a ROS-1 resistance mechanism in a case report [129]. Based on autopsy findings, this phenotype change was associated with retinoblastoma-1 (RB1)- and TP53-gene inactivations, and loss of ROS-1-fusion expression [130]. This resistance mechanism is well-described with other oncogenic drivers, such as EGFR mutations or ALK rearrangements [131]. “Secondary” small-cell carcinomas with driver mutations seem less sensitive to platinum–etoposide chemotherapy than “primary” small-cell carcinomas. In the case of secondary *ROS-1* rearrangement, the patient’s tumor responded only to platinum–etoposide chemotherapy. Optimal therapeutic management of histological transformation remains to be determined.

### 7.2. Under Lorlatinib

ROS-1 resistance mechanisms appearing under TKIs other than crizotinib have not been thoroughly examined. Lin et al., analyzed 28 biopsies of ROS-1-positive tumors that had progressed under lorlatinib. Other punctual mutations in the kinase domain were reported, notably G2032K and L2086F mutations (Table 3) [119]. Resistance to crizotinib, entrectinib and lorlatinib was shown in vitro for the ROS-1^G2032K^ variant. For the ROS-1^L2086F^ variant, structural models to predict resistance to crizotinib, entrectinib and lorlatinib suggested potential cabozantinib efficacy [119]. One patient with an L2086G mutation received cabozantinib that controlled the disease for 11 months. Other lorlatinib-resistance mechanisms were also identified, including MET amplification (4%), KRAS^G12C^ mutation (4%), KRAS amplifications (4%), NRAS amplifications (4%), and MAP2K1 mutations (4%). In a phase I trial on taletrectinib, an L2086F resistance mutation was also found in a patient whose tumor was sensitive to cabozantinib [109].

## 8. What Strategy for the Therapeutic Management of ROS-1-Positive NSCLCs?

Despite the development of new molecules with highly promising pre-clinical phase anti-ROS-1 activity, none has yet demonstrated superiority over crizotinib, which remains the first-line reference therapy for metastatic ROS-1-positive NSCLCs [132]. When brain metastases are found, entrectinib is a therapeutic option for crizotinib-naïve patients. At present, the appearance of resistance mechanisms and activation of escape pathways inevitably lead to tumor progression, with a median time to progression of 5.5–20 months, depending on the patient’s characteristics. When NSCLCs progress on crizotinib, the choice of second-line therapy depends on the type of progression and the molecular profile of the resistant tumor. In the case of oligo-progression, stereotaxic radiotherapy delivered to the progression site should be considered, notably for a brain location [132,133]. For diffuse progression, platinum-doublet-based chemotherapy remains the standard. Some authors prescribed second-line therapy adapted to the resistance mechanism: lorlatinib for non-G2032R mutations and platinum-doublet-based chemotherapy for the G2032R mutation [133].

## 9. Conclusions

Targeted therapies are the cornerstone to treat metastatic ROS-1-positive NSCLCs. Crizotinib remains the first-line reference therapy but new molecules, better able to penetrate the blood–brain barrier, such as entrectinib or lorlatinib, have a role to play in the control of brain disease. However, as for other oncogenic drivers, better understanding of the resistance mechanisms is now essential to guide the choice of the optimal therapeutic sequence. New molecules, such as repotrectinib or taletrectinib, are promising, especially to counter the *ROS-1*-G2302R-fusion variant, the predominant resistance mechanism under crizotinib.

The small number of patients in this specific category make devising randomized clinical trials impossible, but orienting patients towards such endeavors remains important for the development or therapies adapted to the molecular profile of each patient.

## Figures and Tables

**Table 1:** Main *ROS-1*-fusion partners in *ROS-1*-positive non-small-cell lung cancers.

Gene	Description	Frequency	Reference
*CD74*	Cluster of differentiation 74 (several subtypes: C6R34, C6R32 C7R32, C3R34)	38–54%	[11]
*EZR*	Ezrin	13–24%	[37]
*SDC4*	Syndecan 4	9–13%	[37]
*SLC34A2*	Solute carrier family-34 member-2 gene	5–10%	[11]
*TPM3*	Tropomyosin-3 gene	3–15%	[14]
*FIG or GOPC*	Fused in glioblastoma (associated with cancers other than NSCLC) or golgi-associated PDZ and coiled-coil motif-containing	2–3%	[38]
*ADGRG6*	Adhesion G protein-coupled receptor G6	1%	[39]
*ANKS1B*	Ankyrin repeat and sterile alpha motif domain containing 1B	1%	[40]
*CCDC6 or CCKC6*	Coiled-coil domain containing 6	1%	[34,41]
*CEP72*	Centrosomic protein 72	1%	[42]
*CLTC*	Clathrin heavy chain	1%	[43]
*FAM135B*	Family with sequence similarity 135 member B	1%	[44]
*FBXF17*	F-box and leucine-rich repeat protein 17	1%	[44]
*FRK*	Src family tyrosine kinase	1%	[40]
*KDELR2, ELP-1 or ERD2.2*	Endoplasmic reticulum protein retention receptor 2	1%	[34]
*SKT*	Human homologue of murine *Skt* (Sickle tail)	1%	[41]
*LIMA (or EPLIN)*	LIM (Lotus-Intel-Microsoft) domain and actin-binding 1	1%	[2]
*LRIG3*	Leucine-rich repeats and immunoglobulin-like domain 3	1%	[14]
*MLL3*	Mixed lineage leukemia	1%	[12]
*MPRIP*	Myosin phosphatase Rho-interacting protein	1%	[45]
*MSN*	Moesin	1%	[2]
*MYH9*	Myosin, heavy polypeptide 9, non-muscle	1%	[34,46]
*MYOC 5*	Myosin-gene family myosin VC	1%	[23]
*RBPMS*	RNA-binding protein with multiple splicing	1%	[47]
*SLC2A4RG*	solute carrier family-2 member-4	1%	[34]
*SLC6A17*	Solute carrier family-6 member-17	1%	[42]
*SLMAP*	Sarcolemma-associated protein	1%	[23]
*SNN*	Stannin	1%	[41]
*SQSTM1*	Sequestosome 1	1%	[40]
*TDP52L1*	Tumor protein D52-like 1	1%	[42]
*TMEM106B*	Transmembrane protein 106B	1%	[8]
*TRG or TFG*	TRK (transketolase-related gene)-fused gene	1%	[41]
*WNK1*	Lysine deficient protein kinase 1	1%	[34,41]
*ZZCCHC8 or ZCCH*	Zinc finger CCHC-type containing 8	1%	[41]

**Table 2:** Summary of clinical trials on tyrosine-kinase inhibitors (TKIs) targeting ROS-1 in patients with *ROS-1*-positive non-small-cell lung cancers.

TKI	Clinical Trial	Phase	*N*	ORR (95% CI)	mPFS (mo) (95% CI)	mOS (mo) [95% CI]	1-Year OS	Grade-3/4 Adverse Events (%)
Crizotinib	PROFILE 1001	Prospective I/II	53	72% (58–83)	19 (15–39)	51 (29–NR)		36%
	EUROS-1	Retrospective	31	80%	9	—	—	—
	AcSé	Prospective I/II	36	47% (30–65)	6 (4–9)	17 (9–33)	—	—
	EUCROSS	Prospective II	34	70% (51–85)	20 [8–NR]	Not reached	83%	24%
	METROS	Prospective II	26	65% (44–82)	23 (15–30)	NR	—	27%
	East Asian	Prospective II	127	72% (63–79)	16 (13–24)	33	83%	25%
	Shanghai	Retrospective	30	87% (73–97)	18 (6–30)	NR	81%	23%
	Beijing	Retrospective	56	84%	15 (11–19)	NR	—	—
	China	Retrospective	168	86%	18	—	—	—
Entrectinib	ALKA-372-001/STRATRK-1/STARTRK-2	Prospective I/II	161	67% (59–74)	16 (11–21)	NR	81%	31% ^a^
Lorlatinib	NCT01970865	Prospective I/II	69	62% (38–82) ^b^ 35% (21–52) ^c^	21 (4–32) ^b^ 9 (5–15) ^c^	—	—	43%
Ceretinib	NCT01964157	Prospective II	32	62% (45–77)	9 (0–22) ^d^ 19 (1–37) ^b^	24 (5–43)	—	37%
Ensartinib	NCT03608007	Prospective II	59	27% (14–41)	—	—	—	25%
Cabozantinib	NCT01639508	Prospective II	—	—	—	—	—	—
Repotrectinib	TRIDENT	Prospective I	—	—	—	—	—	—
Taletrectinib	United States	Prospective I	6	33% ^c^	4 (1–14) ^c^	—	—	26%
	Japan	Prospective I	15	58% ^d^ 67% ^b^ 33% ^c^	—	—	—	—

^a^ Preliminary results based on 53 patients, ^b^ Results for crizotinib-naïve patients, ^c^ Results for crizotinib-resistant patients, ^d^ Results for crizotinib-naïve and -resistant patients. Abbreviations: ORR = objective response rate; mPFS = median progression-free survival; mOS = median overall survival; CI = confidence interval; NR = not reached.

**Table 3:** Sensitivities of resistance mutations appearing in the *ROS-1* kinase domain to the different tyrosine-kinase inhibitors. (red: No anti-tumor activity (in vitro and/or clinical); orange: In vitro anti-tumor activity only at high concentration; green: Anti-tumor activity (in vitro and/or clinical) demonstrated).

Resistance Mutation in the ROS-1 Kinase Domain	Crizotinib	Entrectinib	Ceritinib	Lorlatinib	Brigatinib	Cabozantinib	Repotrectinib	Taletrectinib	Ensartinib	Foretinib
Appearing on crizotinib										
G2032R (33%–41%) Location: solvent front of the kinase hinge Mechanism: steric hindrance										
D2033N (2.4–6%) Location: Solvent front of the kinase hinge Mechanism: modification of electrostatic forces at the exterior surface of the ATP-binding site and reorientation of surrounding residues in the solvent front of the ATP-binding pocket										
L2026M (2.4–6%) Location: Gatekeeper position in the inhibitor-binding pocket										
S1986Y/F (1%) Mechanism: obstruction in the path to the enzyme active site and increased kinase activity										
L2155S (1%) Mechanism: protein malfunction										
L1951R (1%)										
S1886 (1%)										
Appearing on lorlatinib										
L2086F Mechanism: steric hindrance										
G2032K										
References	[119,120,123,124]	[88,119]	[118,124]	[119,124]	[106]	[88,104,124]	[107,124]	[109]	[109]	[124]

## Data Availability

Not applicable.
